# Association study of genetic variants in eight genes/loci with type 2 diabetes in a Han Chinese population

**DOI:** 10.1186/1471-2350-11-97

**Published:** 2010-06-15

**Authors:** Ying Lin, Pengqiu Li, Li Cai, Ben Zhang, Xin Tang, Xuejun Zhang, Ying Li, Yang Xian, Yang Yang, Li Wang, Fang Lu, Xiaoqi Liu, Shaoqin Rao, Ming Chen, Shi Ma, Yi Shi, Mingjing Bao, Jichuan Wu, Yan Yang, Jiyun Yang, Zhenglin Yang

**Affiliations:** 1 Center for Human Molecular Biology & Genetics, Sichuan Academy of Medical Sciences & Sichuan Provincial People's Hospital, Chengdu, Sichuan, China; 2 Institute of laboratory Medicine, Sichuan Academy of Medical Sciences & Sichuan Provincial People's Hospital, Chengdu, Sichuan, China; 3 Department of Internal Medicine, Sichuan Academy of Medical Sciences & Sichuan Provincial People's Hospital, Chengdu, Sichuan, China

## Abstract

**Background:**

At least twenty genes/loci were shown to be associated with type 2diabetes in European original populations. Five of these genes were shown to be associated with type 2 diabetes (T2D) in Chinese populations. The purpose of this study was to replicate the association of genetic vairants in the eight diabetes-related genes/loci with type 2 diabetes in a Han Chinese cohort from western part of China. Nineteen single nucleotide polymorphisms (SNPs) from the eight genes/loci including *TCF7L2, HHEX, CDKAL1, SLC30A8, PPARG, IGF2BP2, KCNJ11*, and *CDKN2A/CDKN2B *were genotyped in 1,529 cases and 1,439 controls in a Han Chinese population using the ABI SNaPshot method. The meta-analysis of the association between rs7903146 in *TCF7L2 *gene and T2D in the Han Chinese was performed.

**Results:**

Among the eight genes/loci examined, we found that four were significantly associated with T2D. Although previous studies showed that the association between the SNP rs7903146 in the *TCF7L2 *gene and T2D was controversial within the Han Chinese population, we have confirmed the significant association between the SNP rs7903146 in the *TCF7L2 *gene and T2D in both this study and the meta-analysis in the population. In addition, we also confirmed that three SNPs (rs1111875, rs7923837 and rs5015480) in *HHEX *, one SNP (rs10946398) in *CDKAL1*, and three SNPs (rs13266634, rs3802177 and rs11558471) in *SLC30A8 *were significantly associated with T2D in the population being studied.

**Conclusions:**

We demonstrated that the variants in *TCF7L2, CDKAL1, HHEX*, and *SLC30A8 *genes are associated with T2D in a Han Chinese population.

## Background

Diabetes is characterized as hyperglycemia that occurs when the pancreas does not produce enough insulin, or when the body cannot effectively use the insulin it produces. Globally, diabetes causes about 5% of all deaths each year; this statistic is likely to increase by more than 50% in the next ten years without urgent action http://www.who.int/diabetes/en. In China, it is estimated that the number of diabetes patients will increase from the previous figure of 20.8 million in 2000, to 42.3 million in 2030 [1]. Type 2 diabetes (T2D) is the most common form of diabetes, which is caused by an interaction of multiple genes and environmental factors.

Previous linkage studies and candidate gene approaches have identified several genes associated with T2DM, such as *CAPN10*, *ENPP1*, *HNF4A*, *ACDC*, *PPARG*, and *KCNJ11 *[2-8]. The *TCF7L2 *gene, initially mapped by a linkage study, is the strongest known T2D association gene in European original populations [9]. Whole genome association studies (WGAS) offer a new approach to gene discovery for T2D. For example, genetic variants in more than 15 genes/loci including *CDKN2A/B, CDKAL1, SLC30A8, IGF2BP2, HHEX/IDE, FTO, KCN1, NOTCH2, CDC123/CAMK1D, ADAMTS9, THADA, TSPAN8/LGR5, JAZF1, MTNR1B *and *IRS *genes, have been showed to be associated with T2D by genome-wide association studies (GWAS) [10-19]. Although more and more T2D associated gene/loci are being identified, the replication study has played a critical role in confirming the reported T2D associated genes/loci, especially within different ethnic populations. Among the T2D genes/loci, single nucleotide polymorphisms (SNPs) in *CDKAL1, CDKN2A/B, IGF2BP2, SLC30A8*, and *HHEX/IDE *genes were also shown to have an association with T2D in two Chinese cohorts [20-23]. The association between rs7903146 in the *TCF7L2 *and T2D in the Chinese population is still considered to be controversial in Han Chinese populations from north, east and south China [24-27]. In this study, we examined the associations of nineteen SNPs in eight T2D genes/loci in a Han Chinese population in western part of China.

## Methods

### Subjects

This study was approved by the Clinical Research Ethics Committee of the Sichuan Academy of Medical Sciences & Sichuan Provincial People's Hospital. All subjects provided informed consent prior to participation in the study. Diabetes patients were recruited in the diabetes clinic at Sichuan Academy of Medical Sciences & Sichuan Provincial People's Hospital. Included were 1,529 diabetic cases and 1,439 non-diabetic controls. Diabetes was diagnosed in accordance with the criteria of the World Health Organization [28]. There was not exposure to glucose-lowering treatment for the controls, controls with a fasting plasma glucose concentration <5.6 mmol were enrolled from the same geographical region. The age of the patients, body mass index (BMI), SBP, DBP, GLU, TC, TG, LDL, HDL, HbA1c, and duration of T2D were recorded. All cases and controls were Han Chinese from Chengdu area of China. The basic characteristics of the cases and controls are listed in Table 1.

**Table 1:** Characteristics of cases and controls for age and ethnicity

Variables	Subject group		p value
	Cases (n = 1529)	Controls (n = 1439)	
Age at test (years)	60.2 ± 10.1	58.1 ± 10.8	<0.001
Gender (male %)	47.8	50.0	0.225
BMI (kg/m2)	23.9 ± 2.7	23.5 ± 2.8	<0.001
SBP (mmHg)	136.1 ± 12.0	123.9 ± 16.0	<0.001
DBP (mmHg)	80.04 ± 6.8	76.7 ± 9.3	<0.001
GLU(mmol/L)	8.3 ± 2.7	4.9 ± 0.6	<0.001
TC (mmol/L)	5.22 ± 0.77	5.17 ± 0.87	0.139
TG (mmol/L)	2.09 ± 1.45	1.45 ± 1.14	<0.001
LDL (mmol/L)	3.10 ± 0.85	3.08 ± 0.81	0.497
HDL (mmol/L)	1.40 ± 0.74	1.41 ± 0.30	0.822
HBA1C (%)	7.97 ± 0.73	/	/
DURATION ( years)	8.73 ± 4.57	/	/

Data are shown as mean ± SD; The p values were obtained by comparison of cases and controls			

### Genotyping

The Han Chinese series of 1,529 diabetic patients was genotyped and allele frequencies were compared to 1,439 ethnicity-matched non-diabetic control subjects by lab personnel blinded to case/control status. The DNA was isolated from white blood cells using the phenol/chloroform and ethanol precipitation method. SNPs in eight genes/loci from the first round of European GWASs were analyzed in this study, most significant SNPs in the GWASs or in the replication studies in East Asians were selected to perform genotyping. Nineteen SNPs in eight genes/loci were genotyped using the ABI SNapShot method (Applied Biosystem, CA, USA). In brief, PCR was performed using specific PCR primers; the specific SNaPshot primer was used for a SNaPshot reaction using the purified PCR products as templates. The SNaPshot reaction products were then analyzed on an ABI 3130 Genetic Analyzer (Applied Biosystem, CA, USA). All PCR and SNaPshot primers are listed in Additional file 1. To confirm the genotyping results, 10% of the samples were randomly selected and re-genotyped by direct sequencing using a BigDye terminator (Applied Biosystem, CA, USA); no more than a 2% discrepancy was observed in all SNPs between the two genotyping methods.

### Statistical Analysis

Continuous variables were described as mean ± std ( ± *s*) or quartiles (*P*_25_, *M*, and *P*_75_) for data with and without normality, respectively, and were tested by a Student *t*-test. Categorical variables such as gender (male vs. female) were analyzed using a *Chi*-square test.

We tested the Hardy-Weinberg equilibrium (HWE) for each *SNP *separately in both the case and control populations by using the Fisher's exact method, as reported by Emigh, et al. [29]. Linkage disequilibrium (*LD*) coefficients (*r*^2 ^and *D*') were computed by using Haploview 4.1 http://www.broad.mit.edu/haploview.

A standard *chi*-square test with a 1-degree-of-freedom (df) was used to calculate the differences of allele frequencies for each *SNP *between the case and control group. Odds ratios (ORs) with 95 percent confidence intervals (*CI*s) were assessed for the risk allele of each SNP based on a multiplicative model. For the genotypes, we tested a series of genetic models including additive, dominant/recessive for the *SNP*s with a *p *value of < 0.05 of allelic, trend test by using unconditional logistic regression with adjustment for age, gender, and body mass index (BMI).

SAS 9.1 (SAS Institute Inc., Cary, NC, USA) was used to process the data. Results were confirmed by R 2.7.2 http://cran.r-project.org/; a two-sided P value < 0.05 was considered statistically significant unless stated otherwise. Different genetic models, including dominant and recessive model, were evaluated through pearson *X*^*2 *^test by comparing genotypic counts between cases and controls. Allelic p value is obtained by comparing the allele frequency difference in cases and controls under the assumption of a multiplicative model. An additive model is tested using Armitage's test for a trend [30].

### Meta-analysis of the association between rs7903146 in the TCF7L2 gene and T2D in the Han Chinese population

We obtained the data for rs7903146 in the *TCF7L2 *gene in Han Chinese populations by searching Pub Med using key words of TCF7L2, Han Chinese, and Diabetes [21,24,25,31]. Additional four association studies representing four different parts of China were included in this meta-analysis. The data was then combined with our data in this study and the p value and OR were calculated under the assumption of a multiplicative model. A total of 3203 cases and 3109 controls of Han Chinese population for rs7903146 in the *TCF7L2 *gene were included in this meta-analysis (Table 2).

**Table 2:** Meta-analysis of rs7903146 in *TCF7L2 *with T2D in the Han Chinese populations

			Genotyping count								Allele frequency		Multiplicative model	
			
Authors	Samples collected	References	Cases				Controls				Risk allele (T)			
					
			Total N	CC	CT	TT	Total N	CC	CT	TT	Case	Control	p value	OR (95% CI)^b^
Ren et al	Beijing (North China)	24	481	438	41	2	491	463	26	2	0.047	0.03	0.063	1.93 (1.13-3.49)
Ng et al	Hong Kong (South China)	27	433	408	24	1	419	399	20	0	0.031	0.024	0.17	1.29 (0.90-1.87)
Chang et al	Taiwan (East China)	25	760	724	35	1	760	716	44	0	0.024	0.029	0.36	0.81 (0.50-1.31)
Lin et al	Chengdu (West China)	This study	1529	1348	178	3	1439	1328	107	4	0.060	0.040	0.00038	1.54 (1.21-1.95)
Combined all	Han Chinese in China		3203	2918	278	7	3109	2906	197	6	0.046	0.034	0.00058	1.37 (1.15-1.65)

N, Number; b, OR, odds ratio, CI, confidence interval														

## Results

We genotyped nineteen representative SNPs in eight potential T2D genes/loci a Han Chinese population, comprised of 1,529 cases and 1,429 controls. All SNP were within Hardy-Weinberg equilibrium in controls (P > 0.05, Table 3). Linkage disequilibrium (LD) analysis of SNPs genotyped for each gene/locus demonstrated that SNPs in *SCL30A8 *and *PPARG *genes, but not in other genes, were at the same LD, respectively (Table 3).Three SNPs in the *SCL30A8 *gene were at the same LD with *D*' of 0.95 to 0.99, but they were not completely at the same LD because the *r*^2 ^was less than 0.8 from 0.55 to 0.67. Three SNPs in the *PPARG *gene were also showed at the same LD with *D*' from 0.7 to 0.89, but they were not strongly at the same LD because of lower *r*^2 ^(0.022 to 0.38) (Table 3).

**Table 3:** Association between type 2 diabetes and 19 SNPs in the eight genes/loci

Gene	SNP	No.	LD^a ^D'(r^2^)	P value for HWE^b^		Allel Frequency		Additive model						
				
				Cases	Controls	Cases	Controls	Trend p value	Adjusted p value	OR^c ^(95% CI)^d^	Adjusted p value	OR (95% CI)	Adjusted p value	OR (95% CI)
*TCF7L2*	rs6585205 (T)	1	1 vs 2: 0.28 (0.0070)	0.54	0.31	0.43	0.39	0.003	0.055		4.0 × 10^-4^	1.31 (1.13, 1.53)	0.287	1.11 (0.92, 1.35)
	rs7903146 (T)	2	1 vs 3: 0.72 (0.33)	0.25	0.24	0.06	0.04	3.6 × 10^-4^	0.007	1.54 (1.19, 1.99)	0.001	1.58 (1.25, 2.03)	0.691	1.41 (0.40, 5.02)
	rs11196218 (A)	3	2 vs 3: 0.28 (0.01)	0.26	0.60	0.29	0.30	0.520	9.880					
*HHEX*	rs1111875 (G)	4	5 vs 4: 0.39 (0.11)	0.11	0.32	0.32	0.28	0.001	0.016	1.11 (0.97, 1.27)	0.005	1.23 (1.07, 1.42)	0.011	1.43 (1.08, 1.89)
	rs7923837 (G)	5	5 vs 6: 0.53 (0.18)	0.24	0.99	0.30	0.25	1.4 × 10^-5^	2.7 × 10^-4^	1.08 (0.93, 1.25)	8.6 × 10^-6^	1.39 (1.20, 1.61)	0.062	1.30 (0.99, 1.72)
	rs5015480 (C)	6	4 vs 6: 0.69 (0.21)	0.69	0.41	0.21	0.17	0.001	0.027	1.20 (0.96, 1.54)	3.3 × 10^-4^	1.32 (1.14, 1.54)	0.657	1.08 (0.75, 1.59)
*CDKLA1*	rs736425 (C)	7	8 vs 9: 0.55 (0.056)	0.48	0.93	0.07	0.07	0.782	14.849					
	rs10946398 (C)	8	7 vs 8: 0.11 (0.0011)	0.79	0.36	0.45	0.37	1.2 × 10^-9^	2.2 × 10^-8^	1.36 (1.20, 1.55)	2.7 × 10^-6^	1.44 (1.24, 1.68)	6.3 × 10^-9^	1.78 (1.46, 2.17)
	rs4712527 (G)	9	7 vs 9: 0.086 (0.0020)	0.07	0.52	0.22	0.21	0.270	5.132					
*SCL30A8*	rs13266634 (C)	10	10 vs 11 0.99 (0.55)	0.13	0.08	0.59	0.54	1.4 × 10^-4^	0.003	1.37 (1.20, 1.56)	0.005	1.32 (1.09, 1.60)	0.001	1.31 (1.11, 1.53)
	rs3802177 (C)	11	10 vs 12 0.95 (0.64)	0.96	0.15	0.60	0.53	9.9 × 10^-8^	1.9 × 10^-6^	1.33 (1.18, 1.52)	0.007	1.29 (1.07, 1.57)	1.2 × 10^-8^	1.58 (1.35, 1.84)
	rs11558471 (A)	12	11 vs 12 0.96 (0.67)	0.96	0.86	0.58	0.52	3.5 × 10^-6^	6.6 × 10^-5^	1.25 (1.11, 1.41)	1.7 × 10^-4^	1.41 (1.18, 1.69)	1.5 × 10^-4^	1.35 (1.16, 1.59)
*PPARG*	rs180282 (G)	13	13 vs 14: 0.87 (0.079)	0.30	0.78	0.06	0.05	0.394	7.482					
	rs12636454 (C)	14	14 vs 15: 0.89 (0.38)	0.95	0.81	0.32	0.33	0.523	9.928					
	rs11128597 (A)	15	13 vs 15: 0.70 (0.022)	0.14	0.43	0.48	0.46	0.296	5.618					
*IGF2BP2*	rs4402960 (T)	16		0.46	0.53	0.23	0.22	0.176	3.335					
*KCNJ11*	rs5215 (C)	17		0.21	0.28	0.42	0.41	0.225	4.277					
*CDKN2A/*	rs564398 (C)	18	18 vs 19 0.12 (0.0011)	0.00	0.27	0.01	0.01	0.627	11.913					
*CDKN2B*	rs10811661 (C)	19		0.26	0.56	0.05	0.05	0.880	1.670					

a: Linkage disequilibrium (LD), b: Hardy-Weinberg equilibrium, c: Odds ratio (OR), d: Confidence interval (CI).

SNPs in four genes including *TCF7L2*, *CDKAL1, SLC30A8 *and *HHEX/IDE *showed significant association with T2D in the studied cohort even after a stringent Bonferroni correction (adjust p values < 0.05, Table 3). One SNP (rs7903146) in the *TCF7L2 *gene, and three SNPs (rs1111875, rs7923837 and rs5015480) in the *HHEX *gene showed significant association with T2D both in multiplicative and dominant models (adjusted p < 0.027, Table 3). In addition, one SNP (rs10946398) in the *CDKAL1 *gene and three SNPs (rs13266634, rs3802177 and rs11558471) in the *SLC30A8 *gene showed significant association with T2D in multiplicative, dominant and recessive models (adjusted p < 0.0026, Table 3). The rs10946398 in *CDKAL1 *had the strongest association with T2D, the frequency of allele T was 0.45 in case subjects and 0.37 in control subjects. Individuals with risk allele T of rs10946498 conferred a 1.78 fold (95% CI: 1.46~2.17) of increased likelihood of T2D (adjust p = 6.26 × 10^-9^) with recessive model (Table 3). In the studied cohort, no association with T2D was found in the typed SNPs in the four potential T2D genes/loci, including *PPARG, IGF2BP2, KCNJL1*, and *CDKN2A/B *(Table 3).

Meta-analysis of rs7903146 in the *TCF7L2 *gene with T2D in four cohorts of Han Chinese populations composed of 3,203 cases and 3,109 controls also supported that rs7903146 in the *TCF7L2 *was significantly associated with T2D in the Han Chinese population (trend p = 6.2 × 10^-4^) with OR of 1.37.

## Discussion

The Chinese population accounts for approximately 20 percent of the world's population. The replication study of T2D genes/loci in this population has expanded the genetic investigation of T2D in a large ethnic group. Previous association studies of genetic variants in the eight genes and T2D of the Han Chinese populations were from Hong Kong (south), east and north China. The Chinese population of this study was taken from a Han Chinese population in western part of China. Because significant differences exist among Han Chinese subpopulations in China [32,33], the population in this study represented a different type of Han Chinese subpopulalation for T2D association study of genetics, which was also supported by the allele frequency differences of SNPs in the significantly associated T2D genes among Han Chinese subpopulations from different parts of China, for instance, the C allele frequency of rs1326634 in *SCL30A8 *gene was 0.59 in cases and 0.54 in controls in this study compared to 0.42 in cases and 0.47 in controls in the study by Xiang et al. [23]. Therefore, this study not only replicated, but also complemented the previous studies of genetic variants and T2D in the Chinese population.

Although rs7903146 in *TCF7L2 *was confirmed as the strongest T2D genetic variant in European original populations [14], the association of rs7903146 with T2D in east Asian populations, especially in China, remains unclear due to the low frequency of the risk allele of rs7903146 (<5%). Miyake et al. demonstrated that SNP rs7903146 in the *TCF7L2 *gene was significantly associated with T2D in the Japanese population; the adjusted p value was 0.0011 in a study composed of 1,921 cases and 1,696 controls [14]. In another study dealing with the Japanese population, there was a marginal association between rs7903146 and T2D. The p value was 0.0485 in a cohort composed of 1,630 cases and 1,064 controls [34]. Although Chang, et al. did not find a signifcant association between rs7903146 and T2D in Taiwan Han Chinese populaiton study that included a p value of 0.36 (a study of 760 cases and 760 controls) [25], Maggie, et al. showed that rs7903146 was significantly associated with T2D in a Hong Kong Chinese population; the p value was 0.038 with 433 cases and 419 controls [26]. Ren, et al. also indicated that a trend association between rs7903146 and T2D with a p value of 0.063 in a study of 481 cases and 491 controls [24]. Our results further demonstrated that rs7903146 in the *TCF7L2 *gene was significantly associated with T2D in the Han Chinese population in mainland China; the T risk allele of rs7903146 conferred a 1.58 fold increasing the likelihood of having T2D, as compared with individuals who do not carry any of the four risk alleles (adjusted p = 1.0 × 10^-3^, dominant model). This results was also supported by the meta- analysis of the association between rs7903146 in *TCF7L2 *gene and T2D in the four Han Chinese population(Table 2) and in the East Asian populations [31]. Given the factor of low MAF (minor allele frequency) of rs7903146 (<5%), and the 5.5% T2D prevalence in adults in China [26], a power calculation using an additive genetic model showed that approximately 1,500 cases and 1,500 controls would be necessary to achieve 80% power for rs7903146. This indicated that the sample size in some of the previous studies was underpowered in evaluating the association of rs7903146 and T2D in the Chinese populations [24,25,27]. Nevertheless, all studies redarding the association of *TCF7L2 *and T2D in Chinese populations indicated that other different genetic variants in the *TCF7L2 *gene showed a significant association with T2D in Chinese populations [20,24-26]. We also confirmed that another SNP rs6585205 in *TCF7L2 *gene was significantly associated with T2D in the studied cohort with an odd ratio of 1.31 (adjust p = 4.0 × 10^-4^, dominant model). Although it is certain that TCF7L2 plays an important role in the development of T2D in east Asian populations, it appears that the contribution of TCF7L12 to T2D development in east Asian populations is not as strong as that in Caucasian populations.

Consistent with previous findings [20,21,23,35], we also confirmed that SNPs in the *CDKAL1*, *HHEX*, and *SLC30A8 *genes showed a significant association with T2D in the Han Chinese population being studied. In this study, we found a significant association between all three SNPs typed, including rs1111875, rs7923837 and rs5015480, in the *IDE*-*KIF11*-*HHEX *region, and T2D in the Chinese population. Among the three SNPs in this region, rs7923837 showed the most significant association with T2D with an odds ratio of 1.39 (adjusted p = 8.62 × 10^-6^, dominant model). The association is similar to that previously reported in Chinese populations [21], which further confirmed that the variants in this region play an important role in T2D for different races. However, the frequencies of risk alleles in the three SNPs were much lower in east Asian populations including China and Japan, than those reported in European original populations.

Among the four T2D associated genes, SNPs in *CDKAL1 *and *SLC30A8 *showed the most significant association with T2D in this study, and the significant association between the SNPs of both genes and T2D was observed all three models (multiplicative, dominant and recessive models) being tested. Only one SNP rs10946398 among the three SNPs we typed in *CDKAL1 *showed a significant association with T2D in this study, however this SNP showed to have the most significant association with T2D in all three models (multiplicative, dominant and recessive models) being tested among all nineteen SNPs we typed, indicating that the *CDKAL1 *gene may contribute more than the other three T2D-related genes in the development of T2D in Chinese patients being studied. All three SNPs, including rs13266634, rs3802177 and rs11558471 in the *SLC30A8 *gene were strongly associated with T2D in this study; however, rs3802177 had the most significant assocaition with T2D among the three SNPs with an adjusted p value of 1.22 × 10^-8 ^and an odd ratio of 1.58 when a recessive model was tested. The *SLC30A8 *gene plays the second important role among the four genes/loci in the development of T2D in Chinese Han population among the four T2D-related genes/loci in the study. However, these pre-mature conclusions require further investigaton in different Han Chinese populations.Although we could not replicate the significant association in the SNPs in the *PPARG*, *IGF2BP2*, *KCNJ11*, *EXT2*, *CDKN2A/B*, and *LOC387761 *genes with T2D, which can be explained by investigating a different population in our study. In addition, we cannot exclude the possibility of the association of other SNPs in these genes/loci with T2D in the Han Chinese population. Further studies regarding other SNPs for these genes in large Chinese populaiotns are needed to answer these questions. Because T2D is one of the most common diseases, and there are many genes invloved in the development of this disorder, each gene represents a relatively small risk or protection when they are used to assess a disease threat in a patient.

## Conclusions

We demonstrated that the genetic variants in *TCF7L2, CDKAL1, HHEX*, and *SLC30A8 *were significantly associated with T2D in a different Han Chinese population from those of previous studies, further indicating that these gene/loci may play an important role in the development of T2D in the Han Chinese population. We further confirmed that *TCF7L2 *rs7903146 was significant association with T2D in the Han Chinese population.

## Abbreviations

TCF7L2: Transcription factor 7-like 2; HHEX: Hematopoietically expressed homeobox; CDKAL1: CDK5 regulatory subunit associated protein 1-like 1; SLC30A8: Solute carrier family 30 (zinc transporter), member 8; PPARG: Peroxisome proliferator-activated receptor gamma; IGF2BP2: Insulin-like growth factor 2 mRNA binding protein 2; KCNJ11: Potassium inwardly-rectifying channel, subfamily J, member 11; EXT2: Exostoses (multiple) 2; CDKN2A/CDKN2B: Cyclin-dependent kinase inhibitor 2A/cyclin-dependent kinase inhibitor 2B; CAPN10: Calpain 10; ENPP1: Ectonucleotide pyrophosphatase/phosphodiesterase 1; HNF4A: Hepatocyte nuclear factor 4, alpha; ACDC: Pcyl-CoA dehydrogenase; PCR: Polymerase Chain Reaction; SBP: Systemic blood pressure; DBP: Diastole blood pressure; GLU: Glucose; TC: Total cholesterol; TG: Total triglyceride; LDL: Low-density lipoprotein; HDL: High-density lipoprotein; HBA1C: Glycosylated haemoglobin; ADAMTS9: ADAM metallopeptidase with thrombospondin type 1 motif, 9; THADA: thyroid adenoma associated; TSPAN8/LGR5: transmembrane 4 superfamily member 3/leucine-rich repeat-containing G protein-coupled; JAZF1: JAZF zinc finger 1; MTNR1B: melatonin receptor 1B; IRS: insulin receptor substrate 1; LD: Linkage disequilibrium; HWE: Hardy-Weinberg equilibrium

## Competing interests

The authors declare that they have no competing interests.

## Authors' contributions

Designed the study: YL and ZY; collected samples and experiments: YL, PL, LC, XT, XZ, YL, YX, YY, LW, FL, XL, SR, MC, SM, YS, MB, JW, YY, JY and ZY; performed the data analysis: BZ, YL and ZY; writing the manuscript: ZY. All authors read and approved the final manuscript.

## Pre-publication history

The pre-publication history for this paper can be accessed here:

http://www.biomedcentral.com/1471-2350/11/97/prepub

## Supplementary Material

Additional file 1 **Supplementary Table S1**. PCR and SNaPshot primers of SNPs in the ten potential T2D genes/loci Click here for file
